# Psychophysiological effects of traditional cycling, virtual reality-enhanced cycling, and passive virtual reality exposure in young adults: A controlled within-subject study

**DOI:** 10.1371/journal.pone.0343812

**Published:** 2026-03-09

**Authors:** Saad A. Alhammad, Abdulmajeed Y. Alhozaimi, Abdulrahman A. Kateeb, Abdulrhman S. Alghamdi, Maha F. Algabbani, Fahad S. Algarni, Mohammed Alrashidi, Mohammed A. Almeshari, Ayedh Alahmari, Shahad M. Almohsen, Khalid S. Alwadeai

**Affiliations:** 1 Department of Rehabilitation Health Sciences, College of Applied Medical Sciences, King Saud University, Riyadh, Saudi Arabia; 2 Department of Physical Therapy, College of Medical Rehabilitation Sciences, Taibah University, Al-Madinah Al-Munawarah, Saudi Arabia; 3 Institute of Inflammation and Ageing, University of Birmingham, Edgbaston, Birmingham, United Kingdom; George Mason University, UNITED STATES OF AMERICA

## Abstract

**Background:**

Virtual reality (VR) offers a promising tool to enhance engagement in physical activity, but the independent and combined effects of VR on physiological and psychological responses remain underexplored in young adults.

**Aim:**

To compare acute psychophysiological responses to traditional cycling, VR-enhanced cycling, and passive VR exposure (VR-noEx) in healthy young adults.

**Methods:**

In this randomized, counterbalanced within-subject study, 60 healthy university students aged 18 years or older completed three 10-minute sessions with 10-minute seated rest between conditions: (1) traditional cycling, (2) cycling with VR, and (3) VR-noEx (VR with no exercise). Outcome measures included self-efficacy, enjoyment, perceived exertion, heart rate (HR), systolic and diastolic blood pressure (SBP, DBP), and respiratory rate (RR). Data were analyzed using repeated-measures ANOVA with post-hoc Bonferroni corrections, and effect sizes were calculated (Cohen’s d).

**Results:**

Both cycling conditions significantly increased HR (33–35 bpm), SBP (14–17 mmHg), and RR (5.8–6.5 breaths/min) compared to rest (all p < .001), with no significant differences between VR cycling and traditional cycling. VR-noEx did not significantly alter HR or BP relative to rest. VR cycling produced higher self-efficacy and enjoyment than other conditions (p < .05), with small-to-moderate effects and comparable cardiovascular activation.

**Conclusion:**

VR-enhanced cycling improves self-efficacy and enjoyment without reducing cardiovascular activation, whereas passive VR alone does not confer exercise benefits, suggesting VR-assisted exercise enhances positive psychological responses relevant to physical activity engagement.

## Introduction

Physical inactivity is a major public health concern worldwide, contributing substantially to the burden of noncommunicable diseases and reduced quality of life [1]. University students are particularly vulnerable to declines in physical activity, as the transition to higher education often introduces greater academic demands, increased screen time, and reduced engagement in structured exercise. These lifestyle changes frequently lead to more sedentary behavioral patterns during young adulthood. In Saudi Arabia, the trend is even more marked: studies have shown significant reductions in physical activity during college years, with students citing barriers such as limited time, low motivation, insufficient access to appealing exercise modalities, and contextual factors related to climate, campus infrastructure, and sociocultural norms [2]. These challenges highlight the need for innovative and engaging interventions that can sustainably promote physical activity among young adults in this region.

Virtual reality (VR)–based exercise has emerged as a compelling approach to enhance exercise motivation and engagement. As immersive technologies continue to advance within the broader landscape of digital environments—including the growing metaverse—VR offers unique opportunities to transform the exercise experience [3]. By delivering rich multisensory input, VR environments can redirect attention away from discomfort or exertional cues, increase enjoyment, and evoke more positive affective responses during physical activity. Empirical evidence supports these benefits, showing that VR-enhanced cycling improves enjoyment and attentional focus [4], while subsequent work identified embodiment, presence, and interactivity as distinct components that modulate psychological responses during VR-based exercise [5].

Moreover, VR’s physiological impact appears to depend on the fidelity of sensory and biomechanical cues. For instance, a study [6] showed that congruence between virtual terrain and actual pedaling effort influences perceived exertion and respiratory responses. Extending this line of inquiry, a recent study [7] demonstrated that incongruent VR cycling—where virtual gradients were decoupled from mechanical resistance—increased perceived effort and mean arterial pressure despite identical workloads. Such findings highlight that VR does not merely change the aesthetic context of exercise; it can meaningfully modulate psychophysiological responses depending on its design characteristics.

Beyond laboratory-based studies, VR has shown promise across clinical and rehabilitative settings. Findings indicate improvements in motor learning [8], balance and gait among amputees [9], and emotional engagement in exposure-based therapies [10], demonstrating VR’s capacity to influence psychological and physiological processes across diverse applications. Acute VR-exercise studies also report enhanced enjoyment, greater work rate, and more positive affective profiles compared with non-VR equivalents [11], further supporting the potential of VR to enrich exercise experiences in healthy populations.

Despite the growing evidence base, significant gaps remain. Most VR-exercise research has focused on Western populations, raising concerns about cultural generalizability—particularly for Middle Eastern or Saudi university students whose physical activity behaviors are shaped by unique environmental, cultural, and gender-specific factors. Additionally, most existing research comparing virtual reality–enhanced exercise with traditional exercise shows positive effects of VR programs, but the overall quality, quantity, and sample size of studies are limited, and few have isolated the specific contribution of VR immersion itself beyond comparison with traditional exercise alone [12]. Few studies have directly compared immersive VR, non-immersive VR, and traditional cycling within a single within-subjects design, with most research focusing on two conditions at a time rather than all three [13]. For example, Liu et al. [14] compared mood states across immersive VR, non-immersive VR, and traditional cycling within a repeated-measures design, highlighting the relative scarcity of comprehensive three-condition comparisons in the literature. This limitation hinders clear attribution of observed psychological and physiological effects to immersion, physical exertion, or their interaction. Moreover, studies integrating cardiovascular, respiratory, and psychological outcomes within a single design remain limited, constraining comprehensive characterization of psychophysiological responses to VR-based exercise.

To address these gaps, the present study employed a within-subjects design among healthy Saudi university students to compare three conditions: (1) traditional cycling, (2) cycling with immersive VR, and (3) VR exposure alone without exercise. By simultaneously evaluating psychological, cardiovascular, and respiratory outcomes across all conditions, the study isolates the independent and combined effects of VR immersion and physical exertion. This approach also provides culturally relevant insights into whether VR-enhanced cycling offers added psychological benefits for young adults in a Middle Eastern university setting. We hypothesized that VR-enhanced cycling would elicit more favorable psychological responses—greater enjoyment and self-efficacy and lower perceived exertion—than traditional cycling, while both cycling conditions would produce greater physiological activation than VR exposure alone.

## Methods

### Study design

This randomized, counterbalanced within-subject study was conducted at King Saud University (KSU), Saudi Arabia, between February and June 2023. This within-subjects design ensured that each participant served as their own control. Ethical approval was obtained from the Institutional Review Board of KSU (E-23–7526) on 13^th^ February 2023, and the study adhered to the Declaration of Helsinki. Written informed consent was obtained from all participants.

### Participants

Sixty healthy young adults aged 18 years or older were recruited. Eligibility required answering “no” to all PAR-Q+ items [15]. Exclusion criteria included chronic or acute disease, medication use, pregnancy, or inability to use a stationary bike independently. Sex distribution: 50% female (n = 30). A priori sample size was determined using G*Power 3.1 for repeated-measures ANOVA, assuming medium effect size (f = 0.25), α = 0.05, power = 0.80, requiring n = 52; we recruited 60 to account for attrition.

### Intervention

Healthy university students were recruited using email invitations, campus posters, and class announcements. Eligible participants who expressed interest were screened for inclusion criteria and scheduled for laboratory sessions at KSU, Riyadh, Saudi Arabia. Each participant completed three 10-minute sessions in a counterbalanced order, with 10-minute seated rests between sessions:

1. Traditional cycling: Using a KETTLER Polo M Exercise Bike (Kettler, Ense-Parsit, Germany)
2. VR-enhanced cycling: Using a VirZOOM VR Bike, Meta Quest 2 headset, and VZFIT app (Meta/Facebook, California, USA)
3. Passive VR exposure without exercise (VR-noEx)

Cycling sessions were performed at 60–70 RPM, with resistance set at level 5 (mid-range), monitored using a Magene S3 + Speed/Cadence Dual Mode Sensor. Participants were instructed to maintain a consistent pedaling speed. All sessions were conducted in a controlled laboratory environment under the supervision of trained health professionals, and participants received standardized instructions before each session.

### Outcome measures

All outcome measures, including psychological, physiological, demographic, anthropometric, and physical activity assessments, were completed under the supervision of trained research staff. The research staff administered the assessments, providing standardized instructions and ensuring that all procedures were conducted consistently and according to protocol.

#### Psychological.

Three psychological domains were assessed post-session: self-efficacy, enjoyment, and perceived exertion. Self-efficacy was measured using a three-item, 5-point Likert scale (1 = strongly disagree to 5 = strongly agree) adapted from Gao et al. (2019) [16], evaluating participants’ confidence in performance and skill acquisition. Enjoyment was assessed using a five-item, 5-point Likert scale adapted from Ortega et al. (2019) [17], with one reverse-coded item (“I usually prefer to watch rather than play this activity”). Perceived exertion was measured using a single-item, 5-point Likert scale distinct from the 10-point Borg scale. All scales were treated as continuous variables, and mean scores were calculated for analysis. The validity of similar self-efficacy and enjoyment measures has been supported in previous studies [18, 19].

#### Physiological.

Heart rate (HR), systolic blood pressure (SBP), diastolic blood pressure (DBP), and respiratory rate (RR) were measured before and after each session using a Geratherm DESKTOP blood pressure monitor (Geratherm Medical AG, Geratal, Germany). RR was determined by counting the number of breaths over 30 seconds by a trained research assistant. Perceived exercise intensity was assessed pre- and post-session using the modified Borg Rating of Perceived Exertion (RPE) Scale, a validated measure correlated with physiological markers such as blood lactate and HR. The scale ranges from 1 to 10, representing four intensity levels: 1 = no exertion, 2–3 = mild, 4–6 = somewhat hard, 7–8 = hard, and 9–10 = maximal exertion. Exercise-specific responses were recorded according to established standards [11].

#### Demographics, anthropometrics, and physical activity.

Participants reported their age and sex to provide demographic information. Anthropometric measurements included height, weight, body mass index (BMI, kg/m²), and body fat percentage. Height was measured to the nearest 0.5 cm using a Seca stadiometer (Seca, Chino, CA). Weight and body fat percentage were assessed using a Tanita BC-558 IRONMAN Segmental Body Composition Monitor (Tanita, Tokyo, Japan), which estimates body fat via bioelectrical impedance, a reliable method for young adults [20]. BMI was calculated by dividing weight in kilograms by the square of height in meters. Muscle mass and fat mass (kg) were also obtained using the body composition analyzer (Medigate, Wonju-si, South Korea).

Baseline physical activity was assessed using the International Physical Activity Questionnaire (IPAQ), which captures self-reported activity across walking, moderate, and vigorous-intensity activities over the previous seven days [21, 22]. Responses were converted to Metabolic Equivalent Task (MET) minutes per week and categorized as low, moderate, or high physical activity. The IPAQ has been validated for use in diverse populations and is appropriate for descriptive profiling of habitual activity [20].

### Statistical analysis

Normality confirmed via the Shapiro-Wilk test. Repeated-measures ANOVA was used to examine differences across conditions, with Bonferroni corrections for post-hoc comparisons. Effect sizes (Cohen’s d) were calculated for within-subject comparisons. Psychological outcomes were analyzed as continuous variables. Significance was set at p < .05. Analyses were conducted using SPSS v25 (Armonk, NY).

## Results

### Participant characteristics

At baseline, the 60 participants were healthy young adults with a mean age of 20.6 ± 1.0 years, a mean height of 172.9 ± 4.9 cm, and a mean weight of 75.9 ± 11.9 kg, yielding an average BMI of 25.4 ± 3.4 kg/m². Twelve participants (20%) wore spectacles. Physical activity levels, assessed via IPAQ, were predominantly moderate (45%), with 33.3% categorized as low and 21.7% as high. Participants had a mean muscle mass of 53.6 ± 5.8 kg (70.6 ± 4.1% of body mass) and fat mass of 19.8 ± 7.2 kg (26.1 ± 5.2% of body mass). Baseline physiological measurements included HR (79.5 ± 11.6 bpm), SBP (127.6 ± 10.4 mmHg), DBP (72.5 ± 9.9 mmHg), and RR (15.4 ± 2.6 bpm). The mean weekly MET score was 1694.4 ± 1561.2 (Table 1).

**Table 1:** Baseline characteristics of study participants (N = 60).

Characteristics	Mean ± SD or n (%)	p-Value
Age (years)	20.6 ± 1.04	–
Height (cm)	172.9 ± 4.9	–
Weight (kg)	75.9 ± 11.9	–
**Wearing spectacles**		<.0001
Yes	12 (20)	–
No	48 (80)	–
**IPAQ category**		0.086
Low	20 (33.3)	–
Medium	27 (45)	–
High	13 (21.7)	–
Body mass index (kg/m²)	25.4 ± 3.4	–
Muscle mass in kg (% body mass)	53.6 ± 5.8 (70.6 ± 4.1)	–
Fat mass in kg (% body mass)	19.8 ± 7.2 (26.1 ± 5.2)	–
Heart rate in (bpm)^**¥**^	79.5 ± 11.6	–
Systolic blood pressure (mmHg)	127.6 ± 10.4	–
Diastolic blood pressure (mmHg)	72.5 ± 9.9	–
Respiratory rate (bpm)^**π**^	15.4 ± 2.6	–
MET	1694.4 ± 1561.2	–

^**¥**^bpm, beats per minute; ^**π**^bpm, breaths per minute; MET, metabolic equivalents; SD, standard deviation; IPAQ, international physical activity questionnaire.

### Psychological outcomes

**Fig 1:**
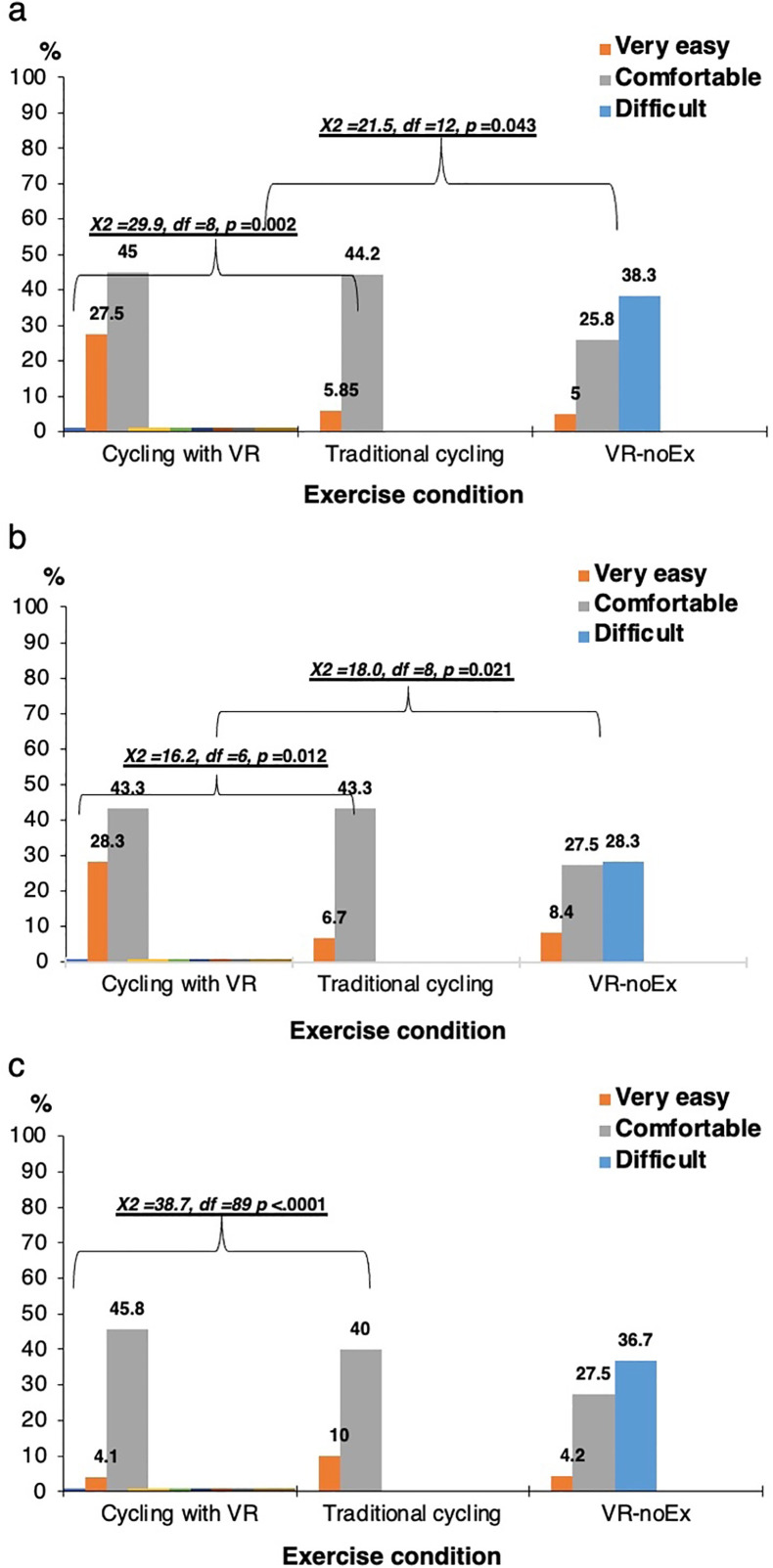
Perceived self-efficacy across exercise conditions.

Participants reported substantially higher self-efficacy during cycling with virtual reality, with the highest proportions rating the activity as comfortable or very easy across all three assessment domains. In contrast, virtual reality without exercise consistently elicited the highest percentage of “difficult” ratings. Significant chi-square tests indicated meaningful differences in perceived difficulty between the three modalities (Panel A: χ² = 29.9–21.5, p ≤ 0.043; Panel B: χ² = 16.2–18.0, p ≤ 0.021; Panel C: χ² = 38.7, p < 0.0001). Overall, combining cycling with virtual reality produced the most favorable self-efficacy profile, whereas virtual reality alone was perceived as the most challenging (Fig 1).

**Fig 2:**
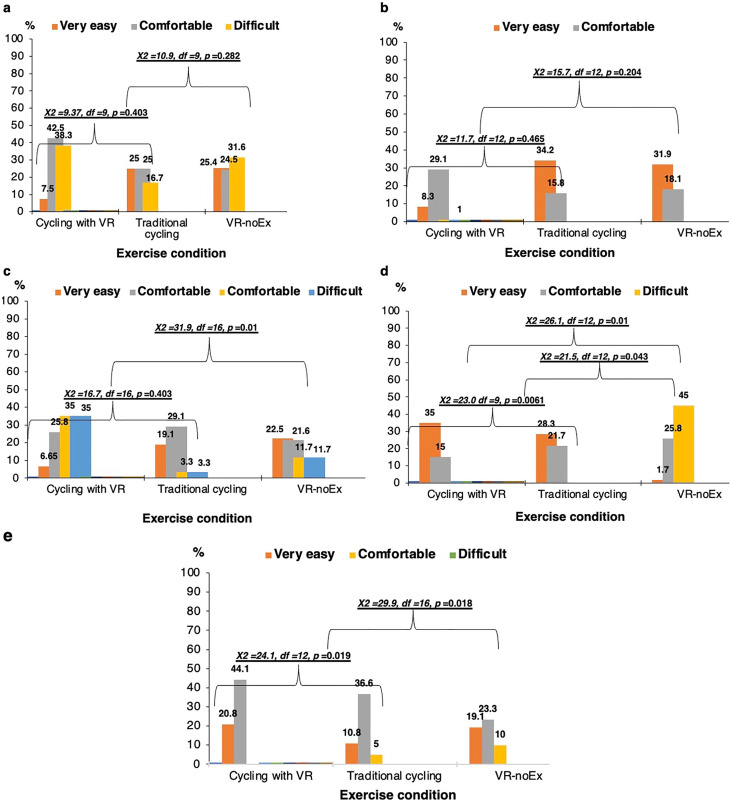
Enjoyment ratings across exercise and virtual reality conditions.

Across all five enjoyment domains, participants consistently rated cycling combined with virtual reality as the most enjoyable modality. This condition showed the highest proportions of “very easy” or “comfortable” responses and the lowest “difficult” ratings across multiple panels. In contrast, virtual reality without exercise showed the highest difficulty ratings in several domains. Significant chi-square tests indicated meaningful differences between the three modalities in panels C, D, and E (χ² range: 23.0–31.9; p ≤ 0.01–0.0061), while panels A and B showed no significant differences. Overall, pairing cycling with virtual reality produced the most favorable enjoyment profile, whereas virtual reality alone was rated least enjoyable (Fig 2).

**Fig 3:**
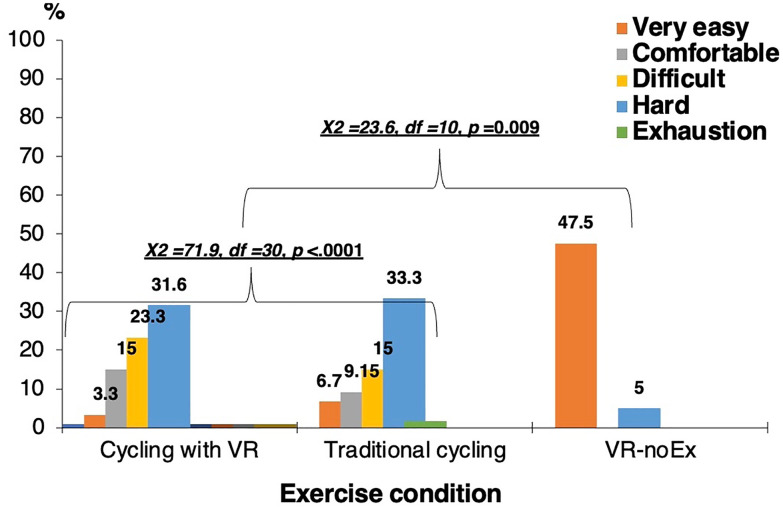
Perceived exertion ratings across exercise and virtual reality conditions.

Fig 3 presents perceived exertion ratings for the three conditions. A significant difference was observed (χ² = 26.8, df = 12, p = 0.01). Most participants rated VR-noEx as “very easy,” whereas traditional and VR cycling were rated as “moderately difficult” or “comfortable.” These results indicate that VR cycling increased perceived exertion similar to traditional cycling but was associated with higher self-efficacy and enjoyment, while VR-noEx was perceived as the easiest condition but offered the least confidence and engagement.

Table 2 compares physiological responses across three conditions: cycling with VR, traditional cycling, and VR without exercise (VR-noEx). VR-noEx elicited significantly lower responses in all physiological measures compared to both active cycling conditions. Specifically, heart rate was lower by 30.4 bpm (d = 1.62), systolic blood pressure by 25.3 mmHg (d = 0.26), diastolic blood pressure by 7.27 mmHg (d = 0.12), and respiratory rate by 6.35 breaths/min (d = 0.53) compared to traditional cycling (all p < 0.0001). Similarly, VR-noEx produced lower values than VR cycling, with heart rate reduced by 31.9 bpm, systolic blood pressure by 22.5 mmHg, diastolic blood pressure by 6.05 mmHg, and respiratory rate by 7.03 breaths/min, demonstrating small to moderate effect sizes.

**Table 2:** Between-condition comparison of physiological parameters across virtual reality and cycling modalities.

Variable	Mean ± SD	MD ± SDD	MD ± SD	Cohen’s *d*
**HR (bpm)** ^**¥**^				
Cycling with VR	114.5 ± 20.3	**Ref.**	–	–
Traditional cycling	112.9 ± 21.3	1.50 ± 13.7^**τ**^	**Ref.**	0.20
VR-noEx	82.6 ± 15.8	31.9 ± 18.7^*^	30.4 ± 21.2^*^	1.62
**SBP (mmHg)**				
Cycling with VR	141.6 ± 14.0	**Ref.**	–	–
Traditional cycling	144.5 ± 12.8	2.90 ± 13.5^**τ**^	**Ref.**	0.20
VR-noEx	119.2 ± 10.1	22.5 ± 13.6^*^	25.3 ± 12.2^*^	0.26
**DBP (mmHg)**				
Cycling with VR	77.9 ± 7.5	**Ref.**	–	–
Traditional cycling	79.1 ± 7.8	1.22 ± 7.0^**τ**^	**Ref.**	0.10
VR-noEx	71.9 ± 6.9	6.05 ± 7.3^*^	7.27 ± 7.6^*^	0.12
**RR (bpm)** ^**π**^		–		
Cycling with VR	21.9 ± 3.9	**Ref.**	–	–
Traditional cycling	21.2 ± 3.5	0.68 ± 3.2^**τ**^	**Ref.**	0.45
VR-noEx	14.9 ± 2.6	7.03 ± 3.9^*^	6.35 ± 3.4^*^	0.53

^**¥**^bpm, beats per minute; ^**π**^bpm, breaths per minute. ^**τ**^ not significant (*p* = 0.399, 0.104, and 0.105, respectively). ^*^Significant (*p* < 0.0001). MD, mean difference; SDD, standard deviation difference; HR, heart rate; SBP, systolic blood pressure; DBP, diastolic blood pressure; RR, respiratory rate; VR, virtual reality; VR-noEx, VR without exercise.

No significant differences were observed between VR cycling and traditional cycling across physiological measures (p = 0.399 to 0.105), with only minor mean differences (e.g., HR: –1.5 bpm), indicating that both active cycling modalities induced comparable cardiovascular and respiratory activation. These results support that VR-noEx does not substantially engage physiological systems, whereas VR cycling provides cardiovascular and respiratory responses equivalent to traditional cycling.

### Physiological outcomes

**Fig 4:**
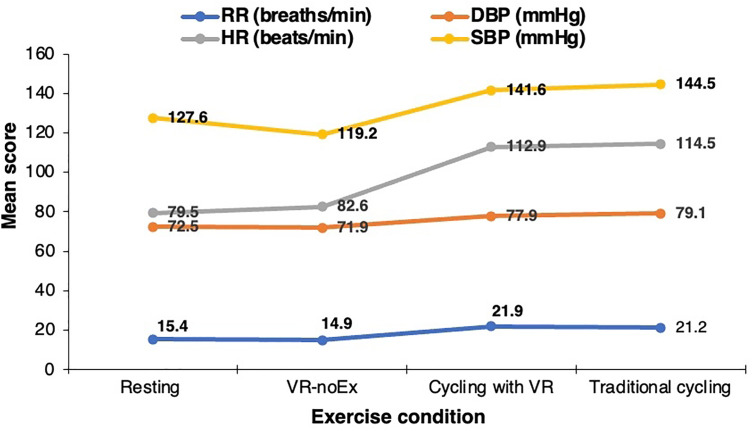
Mean physiological responses across rest, virtual reality without exercise, virtual reality cycling, and traditional cycling sessions.

HR increased markedly from rest (79.5 ± 11.6 bpm) to both exercise conditions: 114.5 ± 20.3 bpm during VR cycling and 112.9 ± 21.3 bpm during traditional cycling. In contrast, VR-noEx showed only a slight, non-significant increase (82.6 ± 15.8 bpm) compared to the resting baseline. SBP rose from 127.6 ± 10.4 mmHg at rest to 141.6 ± 14.0 mmHg with VR cycling and 144.5 ± 12.8 mmHg during traditional cycling, whereas VR-noEx showed a modest reduction (119.2 ± 10.1 mmHg). DBP increased from 72.5 ± 9.9 mmHg at rest to 77.9 ± 7.5 mmHg (VR cycling) and 79.1 ± 7.8 mmHg (traditional cycling), with VR-noEx remaining comparable to rest (71.9 ± 6.9 mmHg). RR increased substantially with both exercise conditions (21.9 ± 3.9 and 21.2 ± 3.5 bpm, respectively), while VR-noEx remained slightly below baseline (14.9 ± 2.6 bpm) (Fig 4).

Heart rate increased by 34.9 ± 18.1 bpm (Cohen’s d = 0.38) with VR cycling and 33.4 ± 18.7 bpm (d = 0.36) during traditional cycling (both p < 0.0001). Systolic BP rose by 14.0 ± 12.6 mmHg (d = 0.17) and 16.9 ± 10.9 mmHg (d = 0.20), and RR increased by 6.50 ± 3.97 bpm (d = 0.29) and 5.82 ± 3.23 bpm (d = 0.27), respectively. DBP increased modestly (5.42 ± 10.1 and 6.63 ± 10.0 mmHg, respectively). In contrast, VR-noEx did not significantly alter HR, DBP, or RR, and only SBP decreased slightly (–8.45 ± 8.60 mmHg, d = 0.10, p < 0.0001), suggesting negligible physiological activation and potential relaxation (Table 3).

**Table 3:** Comparison of physiological parameters during exercise and virtual reality conditions relative to rest.

Variable	Mean ± SD	MD ± SDD	Cohen’s *d*
**HR** (**bpm)**^**¥**^			
Resting	79.5 ± 11.6	**Ref.**	–
Cycling with VR	114.5 ± 20.3	34.9 ± 18.1^*^	0.38
Traditional cycling	112.9 ± 21.3	33.4 ± 18.7^*^	0.36
VR-noEx	82.6 ± 15.8	3.1 ± 14.5^**τ**^	0.03
**SBP** (**mmHg)**			
Resting	127.6 ± 10.4	**Ref.**	–
Cycling with VR	141.6 ± 14.0	14.0 ± 12.6^*^	0.17
Traditional cycling	144.5 ± 12.8	16.9 ± 10.9^*^	0.20
VR-noEx	119.2 ± 10.1	8.45 ± 8.60^*^	0.10
**DBP** (**mmHg)**			
Resting	72.5 ± 9.9	**Ref.**	–
Cycling with VR	77.9 ± 7.5	5.42 ± 10.1^*^	0.07
Traditional cycling	79.1 ± 7.8	6.63 ± 10.0^*^	0.08
VR-noEx	71.9 ± 6.9	0.63 ± 8.79^**τ**^	0.08
**RR (bpm)** ^**π**^			
Resting	15.4 ± 2.6	**Ref.**	–
Cycling with VR	21.9 ± 3.9	6.50 ± 3.97^*^	0.29
Traditional cycling	21.2 ± 3.5	5.82 ± 3.23^*^	0.27
VR-noEx	14.9 ± 2.6	0.53 ± 3.0^**τ**^	0.02

^**¥**^bpm, beats per minute; ^**π**^bpm, breaths per minute. ^**τ**^ not significant (*p* = 0.399, 0.104, and 0.105, respectively). ^*^Significant (*p* < 0.0001). MD, mean difference; SDD, standard deviation difference; HR, heart rate; SBP, systolic blood pressure; DBP, diastolic blood pressure; RR, respiratory rate; VR, virtual reality; VR-noEx, VR without exercise.

## Discussion

This study examined the acute psychophysiological responses of healthy young adults to three activity conditions: traditional cycling, VR-enhanced cycling, and passive VR (VR-noEx). The key findings were that both active cycling conditions elicited significant cardiovascular and respiratory responses compared to rest, whereas VR-noEx did not, and that VR cycling enhanced enjoyment and self-efficacy more than traditional cycling, while VR-noEx was rated as easier but less engaging.

Both VR-enhanced and traditional cycling elicited robust cardiovascular and respiratory activation, with HR increasing by ~33–35 bpm, SBP by ~14–17 mmHg, DBP by ~5–6 mmHg, and RR by ~6 breaths/min, consistent with moderate-intensity exercise expectations. In contrast, VR-noEx produced minimal physiological activation and slightly decreased RR, suggesting a potential relaxation effect. Differences between our results and prior reports of substantial physiological effects from passive VR may reflect methodological variations, including session duration, participant characteristics, or prior VR experience [18, 23, 24]. For example, some earlier studies involved longer sessions or younger, highly VR-experienced participants, which could amplify cardiovascular responses.

VR cycling enhanced self-efficacy and enjoyment compared to traditional cycling and VR-noEx. Both cycling conditions were rated as “comfortable” or “moderately difficult,” whereas VR-noEx was predominantly rated as “very easy.” These findings align with prior research demonstrating that immersive VR can elevate affective responses, motivation, and adherence to exercise regimens [19, 23]. For example, studies [19, 25, 26] reported higher self-efficacy and enjoyment during VR cycling relative to traditional exercise, despite similar physiological intensity. Collectively, these results suggest that VR can enhance the subjective exercise experience without reducing physiological load.

Our findings reinforce that VR-enhanced cycling can improve affective responses while maintaining moderate-intensity physiological engagement, supporting previous work [19, 23, 25, 26]. In contrast, VR-noEx elicited minimal cardiovascular changes, indicating that passive VR cannot substitute for the physiological benefits of active exercise. Some prior studies suggesting large effects from passive VR [12, 27] likely reflect differences in study design, population characteristics, or VR exposure, underscoring the need for careful comparison when interpreting results across studies.

Psychological responses may be influenced by local behavioral patterns, habitual screen time, and prior VR exposure. For example, Saudi university students may engage differently with VR compared to populations studied elsewhere, such as in Poland [22, 28]. Such contextual factors may shape motivational and affective responses, highlighting the importance of considering cultural influences when designing and interpreting VR-based exercise interventions.

### Strengths and limitations

The study provides a controlled comparison of traditional cycling, VR-enhanced cycling, and passive VR within the same participants, allowing for robust within-subject analyses. Several limitations should be noted. First, the sample was relatively small and homogenous (n = 60, young Saudi college students), limiting generalizability. Second, the study was limited to acute responses and did not assess long-term adherence, training adaptations, or motivational changes. Third, baseline physical activity, while measured via IPAQ, was not included as a covariate, which could confound physiological responses [29]. Fourth, exercise intensity was estimated rather than quantified objectively (%HRmax or VO₂), and resistance was fixed at a mid-range level, limiting reproducibility [30]. Finally, minor variability in cadence (60–70 RPM), lack of prior VR experience data, and absence of sex-specific analyses may have influenced outcomes [31]. Despite these limitations, our findings provide meaningful insights into the psychophysiological effects of VR exercise in a Middle Eastern university population and highlight the potential motivational advantages of immersive VR cycling.

## Conclusion

VR-enhanced cycling provides comparable physiological activation to traditional cycling while enhancing self-efficacy and enjoyment. Passive VR without exercise (VR-noEx) does not elicit meaningful cardiovascular or respiratory responses, though it may support relaxation. These findings highlight the potential of immersive VR exercise to optimize both physiological and psychological outcomes, with implications for promoting engagement and adherence in young adults. Future research should examine long-term effects, include diverse populations, and incorporate objective intensity metrics to further refine VR exercise interventions.

## Supporting information

S1 File Dataset. (XLS)
